# Biomarkers for acute kidney injury: is NGAL ready for clinical use?

**DOI:** 10.1186/s13054-014-0680-0

**Published:** 2014-12-10

**Authors:** Claudio Ronco

**Affiliations:** Department of Nephrology Dialysis and Transplantation, International Renal Research Institute of Vicenza (IRRIV), San Bortolo Hospital, Viale Rodolfi, 37, 36100 Vicenza, Italy

## Abstract

The RIFLE (Risk, Injury, Failure, Loss, and End-stage kidney disease) criteria were introduced in 2004, defining the clinical stage of acute kidney injury (AKI) and outcome measures based on serum creatinine, glomerular filtration rate, and urine output. However, a growing body of evidence suggests that these markers are insufficient in drawing an accurate illustration of kidney injury. Indeed, mortality and morbidity remain high in AKI, suggesting that accuracy and speed of patient evaluation are lacking. A great deal of evidence indicates that neutrophil gelatinase-associated lipocalin (NGAL) is a sensitive and specific early marker of various etiological classes of AKI and would be highly valuable in conjunction with existing markers of AKI for better classifying renal injury as well as dysfunction (kidney attack). Improvements in diagnosis, risk identification, stratification, prognosis, and therapeutic monitoring will benefit clinical decision-making in the individualized bundling of therapies and ongoing patient management. In particular, kidney protection and AKI prevention may become feasible if an earlier and more accurate diagnosis is made for AKI. Here, we discuss the opportunity to consider whether NGAL is ready for routine clinical use in a number of etiologies of AKI.

## Early diagnosis of acute kidney injury

In a previous issue of *Critical Care*, Matsa and colleagues [1] suggest that, in patients with no kidney disease prior to admission to the ICU, both plasma (pNGAL) and urinary (uNGAL) neutrophil gelatinase-associated lipocalin have a fair predictive value to diagnose the occurrence of acute kidney injury (AKI) for up to 72 hours. This performance appears to be maintained when such a biomarker is measured at serial time points throughout the ICU stay [1]. This commentary, in conjunction with other lines of evidence in the literature, poses the question of whether NGAL as a biomarker of AKI can be considered ready for clinical routine use [2]. AKI, also called ‘kidney attack’, is defined as an abrupt reduction in kidney function because of multiple causes. Its incidence is increasing in hospitalized patients, especially in conditions of critical illness or aging of the population or both [3-6]. The spectrum of AKI is a continuum that starts with an increased susceptibility and ends with complete failure of the organ. AKI, however, is diagnosed only when a significant number of nephrons are damaged and serum creatinine (sCr) rises above 0.3 mg/dL or a severe oliguria is present. Unlike sCr and urine output, kidney status cannot be comprehensively measured by loss of function alone. SCr concentration may increase slowly, perhaps only following a substantial decrease in kidney function. SCr is also influenced by factors such as age, gender, muscle mass, and nutritional status. AKI can, and should, be diagnosed earlier in order to allow organ protection and prevention of further organ damage. Novel biomarkers such as NGAL seem to represent a suitable possibility to accomplish this task.

What is required for a biomarker to achieve the status of clinical routine test? It should be easy and simple to measure, should be consistent in repetitive measurement, have a rationale for its use, present threshold values that are well documented, be correlated with the presence of illness and with its severity, have a reasonable cost, and finally be measurable in biological fluids that are easily achievable. In this case, uNGAL and pNGAL seem to be both suitable with a small over-performance of urine testing.

AKI is an important outcome measure that prompts therapeutic responses and decisions. Hence, early diagnosis is the key factor for effective prevention and protection. Furthermore, sequential measurements of biomarkers, even in the absence of creatinine rise, may help to identify trends or specific values considered thresholds for the diagnosis. Bagshaw and colleagues [7] demonstrated that patients who developed worsening AKI had a higher serum level of pNGAL compared with those whose AKI did not deteriorate. A systematic review by Haase and colleagues [8] demonstrated the predictive value of NGAL for renal replacement therapy (RRT). For pNGAL levels of greater than 150 ng/mL, the diagnostic odds ratio for subsequent need of RRT was 12.4.

In extended-criteria kidney donors, NGAL has been shown to be an early indicator of kidney graft function and calcineurin inhibitor nephrotoxicity. Ongoing diagnostic applications of NGAL include the assessment of risk, decision-making in single or multiple therapies, and patient monitoring. In cases of suspected sepsis, Kim and colleagues [9] demonstrated the sensitivity of NGAL with sCr in its diagnosis and staging. pNGAL was significantly better associated than sCr with the renal subscore of the Sequential Organ Failure Assessment in critically ill patients with suspected sepsis. Although pNGAL was a sensitive and early marker of AKI within this cohort, it was not possible to distinguish AKI patients in the sepsis cohort, because of the systemic inflammatory nature of the disease [9].

Patient risk assessment is critical to stratify populations and make accurate prognoses as well as identify suitable or harmful therapies for individual patients (theragnostics). This underlines once more the need for the routine clinical availability of early and accurate prognostic indicators. To test the suitability of NGAL as such a tool, clinical trials using point-of-care pNGAL and uNGAL testing should be designed to predict which patients will meet the criteria for AKI. Today, however, criteria for the diagnosis of AKI are subject to change, and the sole increase beyond a given threshold of a biomarker such as NGAL may allow clinicians not only to suspect a risk for AKI but also to make a diagnosis of subclinical AKI or non-creatinine-increase AKI [4-6,10,11].

Elevated NGAL levels have been reported in heart failure, coronary heart disease, and stroke. Outcome in heart failure cases is better predicted by renal markers than cardiac markers, and NGAL has been shown to correlate with cardiovascular disease (CVD) clinical severity. Some studies have shown NGAL to be an independent predictor of major adverse cardiovascular events and mortality; however, this is not yet conclusive. But as an independent marker of CVD, it nonetheless has the potential to offer unique clinical information [4-6,10,11].

Fast and accurate biomarker assays will significantly improve morbidity and mortality by providing diagnosis in hours rather than days. This is particularly pertinent in ongoing patient management and triage decision-making in AKI. The readiness of such technology, and the benefit it confers versus its cost, must be considered.

AKI can have an incidence of up to 7% in the emergency department. There is a limited applicability of the RIFLE (Risk, Injury, Failure, Loss, and End-stage kidney disease) criteria in the emergency setting because of a lack of baseline sCr measures; therefore, the additive value of NGAL testing in AKI, and NGAL and brain natriuretic peptide (BNP) testing in cardiorenal syndrome, becomes remarkable in clinical judgment accuracy as well as in patient risk stratification [12].

NGAL testing has the potential to facilitate rapid decision-making by producing results from small sample volumes in a matter of seconds. This approach is currently being tested with point-of-care testing of NGAL and BNP in the setting of acute fluid resuscitation in severely burned patients [13]. There is an urgent need to prove the viability of these techniques in order for them to be translated into clinical practice.

The next step will be characterized by the feedback to, and early diagnosis of, AKI made by biomarkers. Can we respond in a timely fashion? Do we have specific strategies? Before addressing these questions, we must agree on the fact that a routine use of a test is definitely facilitating its incorporation in the diagnostic armamentarium of a disease or a population. Once we agree on this concept, we will need to recapitulate the pathway that has been made by BNP for heart failure and troponins for acute coronary syndromes.

The need to introduce novel independent biomarkers of AKI into the clinical setting is crucial for earlier diagnosis and improved risk assessment. A parallel problem is the poor understanding of pathophysiology of AKI, which would be improved by the comprehensive characterization of the molecular pathways involved in the propagation of kidney injury.

Some etiological areas may benefit from further work or confirmatory trials in specific populations [1]. However, the development of individual criteria and decision-making frameworks for the etiological variants of AKI is necessary to encompass the variety of factors that can influence clinical decisions. This exercise demands the consensus of experts regarding a proposed protocol for the inclusion of NGAL within evolved RIFLE criteria. At the same time, routine clinical utilization of injury biomarkers will probably be the solution for real advancement in this area.

## Abbreviations

BNP: Brain natriuretic peptide
CVD: Cardiovascular disease
NGAL: Neutrophil gelatinase-associated lipocalin
pNGAL: Plasma neutrophil gelatinase-associated lipocalin
RIFLE: Risk, Injury, Failure, Loss, and End-stage kidney disease
RRT: Renal replacement therapy
sCr: Serum creatinine
uNGAL: Urinary neutrophil gelatinase-associated lipocalin
